# *Hyalomma scupense* (Acari, Ixodidae) in northeast Tunisia: seasonal population dynamics of nymphs and adults on field cattle

**DOI:** 10.1051/parasite/2013012

**Published:** 2013-04-03

**Authors:** Mohamed Gharbi, Mohamed Ettaïeb Hayouni, Limam Sassi, Walid Dridi, Mohamed Aziz Darghouth

**Affiliations:** Laboratoire de Parasitologie, École Nationale de Médecine Vétérinaire, Université de la Manouba 2020 Sidi Thabet Tunisia

**Keywords:** *Hyalomma scupense*, Population dynamics, Seasonality, Tick, Cattle, Tunisia

## Abstract

*Hyalomma scupense* is a two-host tick infesting mainly cattle representing in North Africa the vector of tropical theileriosis (*Theileria annulata* infection), a major tick-borne disease affecting cattle. Any effective control programme of ticks requires a good knowledge of the biology of the target species. In the present study, three cattle farms in northeast Tunisia were surveyed during the activity seasons for adult and nymphs of *Hyalomma scupense*. Several indicators were studied, including chronological indicators, infestation prevalence, infestation intensity and feeding predilection sites of the ticks. The adult ticks were present from mid-June to late November. Nymphs were observed on animals from early September to late November. A large proportion of the ticks were attached in the posterior udder quarters: 41% and 64% of adult ticks and nymphs, respectively. The animals that were heavily infested by adult ticks were also heavily infested by nymphs. Moreover, 17% of adult ticks and 53% of nymphs were present on only 5% of cattle population. These data are important for the success of targeted acaricide application leading to a dramatic decrease of acaricide quantity needed for the treatment. When the preferential sites of attachment are known, the effectiveness of manual removal of ticks can be improved. The presence of highly infested animals is to be considered when any control programme is implemented, since these animals harbour a high proportion of the ticks.

## Abstract (fr)

*Hyalomma scupense* est une tique diphasique qui infeste principalement les bovins, elle représente en Afrique du Nord le vecteur de la theilériose tropicale du bœuf (infection par *Theileria annulata*), une maladie majeure transmise par les tiques affectant les bovins. Tout programme de contrôle des tiques nécessite une connaissance solide de la biologie de la tique cible. Dans le présent travail, trois élevages bovins au Nord-Est de la Tunisie ont été suivis durant la saison d’activité des adultes et des nymphes de *Hyalomma scupense*. Plusieurs indicateurs ont été étudiés, dont les indicateurs chronologiques, la prévalence d’infestation, l’intensité d’infestation et les sites de prédilection des tiques. Les tiques adultes étaient présentes entre mi-juin et fin novembre. Les nymphes ont été observées sur les animaux dès le début de septembre jusqu’à fin novembre. Une large proportion des tiques était attachée aux quartiers postérieurs des mamelles avec respectivement 41 % des adultes et 64 % des nymphes. Les animaux qui étaient lourdement infectés par les adultes l’étaient aussi par les nymphes. De plus, 17 % des adultes et 53 % des nymphes étaient présents chez seulement 5 % de la population des bovins. Ces données sont importantes pour le succès d’une application ciblée des acaricides aboutissant à une diminution drastique des quantités d’acaricides nécessaires pour le traitement. Quand les sites préférentiels d’attachement sont connus, l’effectivité du détiquage manuel peut être améliorée. La présence d’animaux fortement infestés est à prendre en considération pour toute mise en place de programmes de lutte, puisque ces animaux portent une grande proportion des tiques.

## Introduction

Ticks are important vectors for several pathogens (protozoa, viruses, bacteria) which cause severe diseases in humans and animals. In livestock, these arthropods are also directly responsible for production losses affecting animal growth, milk quantity and skin quality [12, 14, 18]. Several tick species are present in Tunisia, *Hyalomma scupense* (syn. *H. detritum*) being one of the more common and economically important species found on livestock, particularly cattle, in several regions of the country [3, 9, 10]. This two-host species exhibits endophilic behaviour [20]. Immature stages of this tick species appear during the autumn on cattle (from early September to late November) and the larvae feed and moult to nymphs on the same host. After detachment from cattle, the engorged nymphs diapause in cracks and crevices of the barn walls where they moult into adults at the end of spring and during the summer [3]. The adults feed on animals during the summer season (from late spring to September with a peak in July) [2], detached engorged females lay their eggs inside the animals’ premises leading to the appearance of a new generation of larvae. In North Africa, *H. scupense* is the vector of the protozoa *Theileria annulata* (Dschunkovsky and Luhs, 1904), the causative agent of tropical theileriosis [20], an important disease of cattle in the region. Furthermore, these tick species also transmit *Coxiella burnetii* (Derrick, 1939) and *Theileria equi* Mehlhorn & Schein, 1998 [23]. In Tunisia, tropical theileriosis represents one of the dominant tick-borne diseases of cattle [4] and the control of *H. scupense* constitutes an important component of any strategy of prevention of tropical theileriosis. In practice, this is achieved using chemical acaricides and/or improvement of cattle enclosures to prevent the off-host tick from finding shelter. Other control measures based on vaccination with tick-exposed and concealed antigens have been developed for several tick species [16], which may represent potential alternatives for controlling ticks of medical and economic importance such as *H. scupense* [1, 5, 16].

A sound knowledge of the phenology of *H. scupense* cattle infestation under typical conditions in Tunisia is essential for optimising existing control measures against this tick as well as the diseases that it transmits. This is also a perquisite for the assessment and application of new control options, such as anti-tick vaccination. Due to the lack of such studies, a field study was carried out within the major endemic region for tropical theileriosis in Tunisia, describing and analysing the characteristics of cattle infestation by *H. scupense* in a number of heavily infested farms.

## Materials and methods

### Animals and study protocol

The present study was carried out in 2006 during the active season of *H. scupense* (from late April to late November) in three neighbouring traditional cattle farms in the village of El Hessiene (northeast of Tunisia, Governorate of Ariana). These farms were chosen for the study due to the expected presence of significant *H. scupense* burdens on cattle. The study site is located within the semi-arid bioclimatic region where tropical theileriosis is enzootic. The climate of this region is characterised by a warm and dry summer (from June to late August) and a wet autumn (from September to late November). The animals are housed in traditionally managed cattle houses, where the walls have not been roughcast and therefore contain many cracks and crevasses providing a suitable habitat for egg laying ticks and nymphs in diapause. Only heifers and cows graze on natural pastures during the morning hours. At the start of the study, all the animals present were identified with numbered ear tags. Tick control measures, such as acaricide application and manual tick removal, were not applied during the study so as not to disturb normal tick activity. In recompense, free veterinary assistance was provided for the three farms throughout the study period by the team of Laboratory of Parasitology (École Nationale de Médecine Vétérinaire de Sidi Thabet). The three farms were visited twice monthly from late April to November. A total number of 14 visits were made for counting the number of adults and nymphs from a total of 95 cattle (36, 26 and 33 animals in each farm respectively). Only animals that were present in the farm for at least 7 visits out of 14 were considered in the analyses. Thus, the results from 86 cattle were utilised, consisting of 34 calves (18 males and 16 females) and 52 adults (3 males and 49 females).

### Global descriptive indicators for tick infestation

#### Determination of species, sex, stages and degree of tick engorgement

The attached ticks were visually counted under bright daylight conditions early in the morning. The sex of the adult ticks was determined on the basis of conscutum development that could be seen with the naked eye.

In order to identify the tick species occurring during the study period, two cows from farm 1 were used as sentinel animals. All the ticks found at each visit on these sentinel cows were collected, preserved in 70% ethanol and examined in the laboratory for species identification according to the key of Walker et al. [23].

The nymphs were identified on the basis of their smaller size while keeping the general morphological features of *Hyalomma* ticks. Species identification was thereafter confirmed on emerged adults obtained after development of the nymphs in the laboratory (27 °C, 95% humidity) according to the key of Walker et al. [23], with ticks being recorded according to their sex and developmental stage. The normalisation of tick burden was made by calculating the natural logarithm of tick numbers: *y* = ln (1 + number of ticks).

#### Seasonal distribution of ticks

The seasonal distribution of counted ticks was established for adult ticks and for nymphs with male and female tick dynamics considered separately.

#### Feeding predilection sites of ticks

Tick feeding predilection sites on cattle were studied in farm 1, whereby the number of ticks feeding on different parts of the body was separately counted. The overall proportions of ticks attached in each anatomical region were determined for adults and nymphal stages of *H. scupense*.

### Effect of animals’ age and sex on tick burdens

The mean number of nymphs and adults per season was processed depending on the age and sex of the animals.

### Animal ranking according to individual tick burdens

The infestation degree (ID) of each animal was calculated for both tick stages as follows [22]:

$${\mathrm{ID}}_{i}=n_{i}/N,$$

where ID_*i*_: infestation degree of the animal *i*, *n*_*i*_: cumulative infestation of the animal *i* and *N*: mean cumulative infestation of all animals present.

During each visit each animal was ranked with regard to tick infestation and the correlation between adult tick burden rank and nymph burden rank was established.

During the visits when the tick population dropped to a residual level, outlier ticks were excluded from the dynamic analysis in order to prevent introduction of bias.

### Statistical analyses

All the data were exported to an Excel^®^ for Windows^®^ spreadsheet. The means were compared using the Student’s *t*-test [19]. In order to compare means among more than two groups, ANOVA was performed, and if significant, the between-group differences identified using the Tukey test, avoiding the alpha inflation. Statistical analyses were conducted using either SPSS 13^®^ for windows^®^ or the SAS 8^®^ for Windows^®^ software packages. A probability below 0.05 was used as a threshold for statistical significance. When parameters were related to the host, they were estimated in both adult and young animals of each sex and both nymphs and adults (male and female). The curves of infestation degrees (ID) were fitted with Best fit 4.5.2^®^ for Windows (Palisade Corporation^®^), the best distribution functions were chosen with regard to the chi-square test for goodness-of-fit.

## Results

### Identification of tick species on sentinel cows

During the study period, a total number of 147 adult ticks were collected from the two sentinel cows from farm 1. Of these, 145 (98.6%) were identified as *H. scupense* and two specimens were identified as *H. marginatum* Koch, 1844. The mean sex ratio (M:F) was 1:0.50 ± 0.88 (range: 1:0.23–1:1.06).

### Tick numbers and seasonal evolution

**Figure 1.**
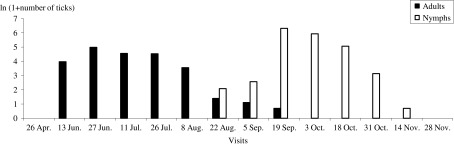
Logarithmic mean intensity of tick infestation of cattle by females and nymphs during the study period.

**Figure 2.**
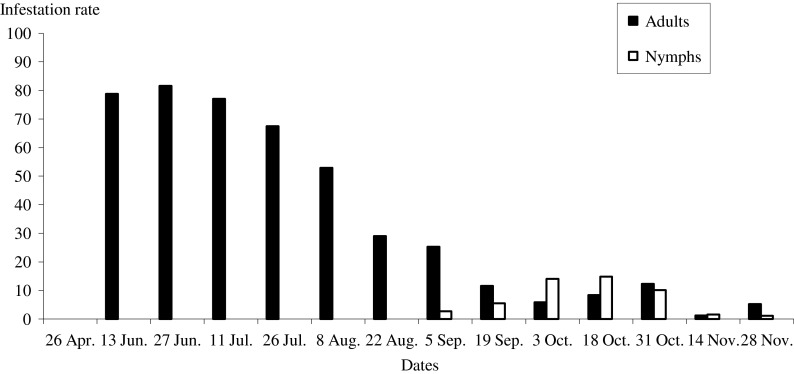
Mean infestation rate of cattle (%) by adults and nymphs during the study period.

A total of 5,305 adult ticks and 4,171 nymphs were counted on 81 of the 86 animals, resulting in a global infestation prevalence of 94.2%. Five calves were found to be uninfested. Both tick stages had marked seasonal activity. Adult ticks were present from June to late November with a peak in late June and this corresponds to the activity peak of females (Figures 1 and 2). The nymphs were observed on animals from late August to late November and during this period, an overlap with adults was observed (Figures 1 and 2).

### Relation of tick infestation with cattle categories

The total numbers of nymphs and adult ticks per season (i.e., the total number of ticks during the summer and autumn season combined) were utilised in the analysis. Cows showed the highest mean intensity of infestation per animal for both nymphs (75.3) and adults (75.8). The lowest intensity of infestation was observed in calves of both sexes (Table 1).

**Table 1.** Mean (standard error) numbers of *Hyalomma scupense* from June to November 2006.

Tick stage	Cows	Bulls	Male calves	Female calves
Adults	75.8^*^ (59.18)	67.7^*^ (77.68)	50.6^*^ (80.07)	12.4^*^ (20.34)
Nymphs	75.3^*^ (100.26)	14.3 (15.63)	3.7^*^ (6.13)	19.5^*^ (51.87)

Asterisks indicate the presence of statistically significant difference in each line for different animal groups.

In order to compare the infestation by adults and nymphs among different animal categories, the mean number of ticks per visit and per animal was compared using the ANOVA test. For adult ticks, the ANOVA was significant (*p* = 0.0001) and the differences identified by the Tukey test. A significant difference in infestation intensity was detected among calves (of both sexes) and cows (*p* < 0.0001) as well as among bulls and cows (*p* < 0.05).

The same statistical analyses were performed for nymphs using an identical approach. The ANOVA test was significant among different animal categories (*p* < 0.005) and the Tukey test revealed a significant difference between calves of both sexes and cows (*p* < 0.005).

**Figure 3.**
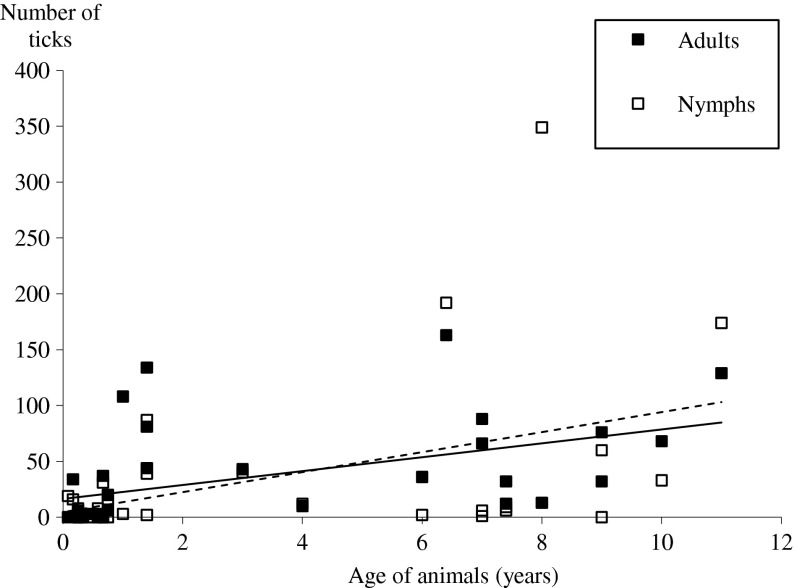
Correlation between age of animals and total tick burdens per animal. Dotted line, correlation for nymphs: *y* = 8.9574*x* + 4.664, *R*^2^ = 0.205. Continuous line, correlation for adults: *y* = 6.1935*x* + 16.521, *R*^2^ = 0.2451.

A positive correlation between the age of the animals and the intensity of adult tick burdens was detected (Pearson correlation = 0.497; *p* < 0.005). A similar positive correlation was also found between nymph tick burdens and the age of animals (Pearson correlation = 0.453; *p* < 0.01; Figure 3).

### Tick attachment sites on the cattle host

The main tick attachment sites on the cattle are presented in Table 2. Cattle body areas with a low skin thickness may represent preferential attachment sites. Thus, the udders harboured 48.76 and 68.74% of the adults and nymphs, respectively. The thighs had burdens of 32.08% and 13.82% of the adult and nymph populations, respectively.

**Table 2.** Mean (standard error) numbers of attached *Hyalomma scupense* adults and nymphs on different predilection sites.

Feeding predilection sites	Adults	Nymphs
Anterior udder quarters	7.54 (0.007)	4.92 (0.007)
Posterior udder quarters	41.22 (0.014)	63.82 (0.015)
Teats	13.15 (0.010)	0.19 (0.001)
Inguinal region	2.73 (0.005)	15.31 (0.011)
Thigh	32.08 (0.013)	13.82 (0.011)
Belly	2.97 (0.005)	0
Axilla	0.32 (0.002)	0
Neck and interscapular region	0	1.95 (0.004)

### Animal ranking according to tick burdens

**Figure 4.**
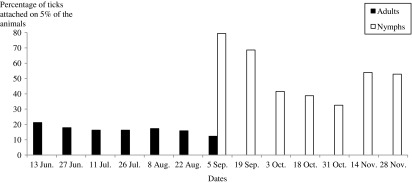
Proportion of adult ticks and nymph counts from 5% of the most infested animals during the survey period. The ratios estimated during early and late periods were not presented since the number of both ticks and infested animals were very low.

The ranking of cattle according to tick burdens showed that a small number of animals were infested by a large number of adults and nymphs. Indeed, 5% of the total cattle population hosted 16.72% of adult ticks (range: 12.28–21.24%) and 52.53% of nymphs (range: 32.59–79.35%; Figure 4).

Furthermore, the most heavily infested animals were predominantly the same, all of them were cows and only one out of 10 was a calf infested by nymphs. “Ticks attractiveness” of cattle was more uniformly distributed in the population, since six cows had the same attractiveness.

**Figure 5.**
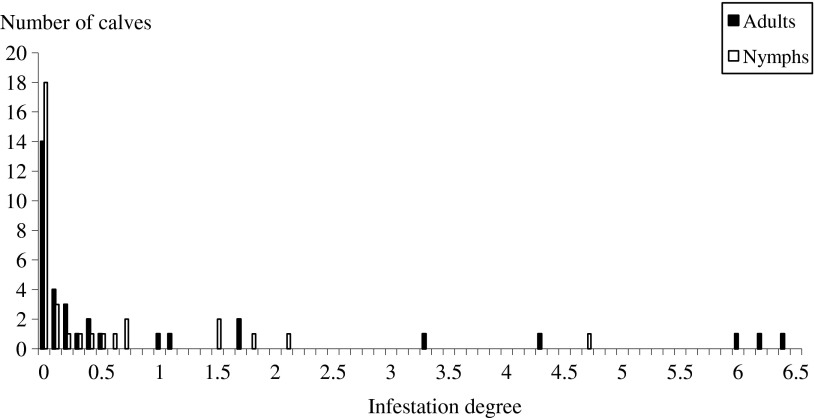
Distribution of infestation degree in calves for adults and nymphs. The infestation degree of the animals is the ratio between the cumulative infestation of animals divided by the cumulative infestation of all the present animals.

**Figure 6.**
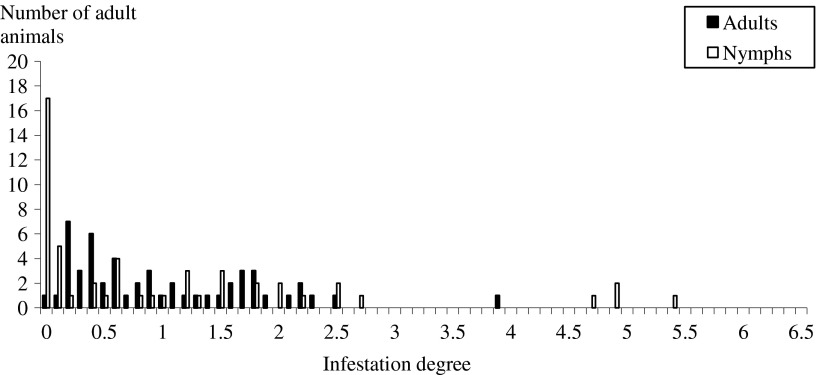
Distribution of infestation degree in adult animals for adults and nymphs. The infestation degree of the animals is the ratio between the cumulative infestation of animals divided by the cumulative infestation of all the present animals.

To avoid bias, the visits where the total population of adult ticks in the three farms was very low (less than 30 ticks) were not considered in the present graph, i.e., 7 visits out of 14 for both adults and nymphs. The ID of calves and adult cattle by adults and nymphs were estimated and plotted (Figures 5 and 6). With the exception of the distribution of adult ticks in adult animals which is triangular, all the curves were fitted to an exponential function. Skewdness of the distributions was lower in adult animals (around 1) than in calves (around 4).

### Correlation between adult and nymph infestation

**Figure 7.**
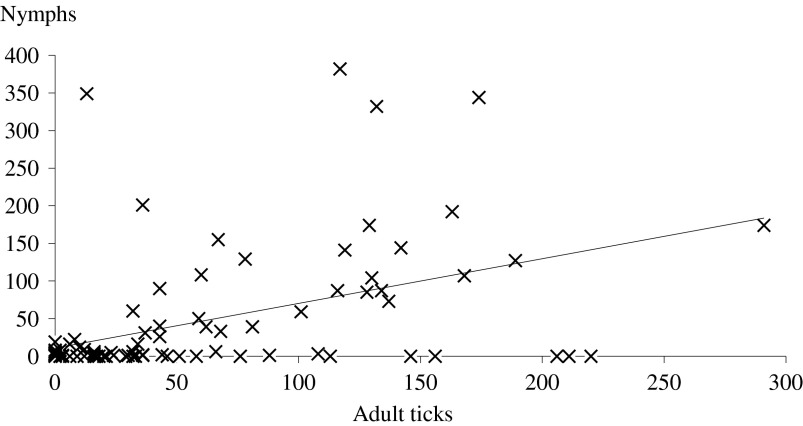
Correlation between overall adult tick and nymphs infestation burdens. *y* = 0.5935*x* + 10.878, *R*^2^ = 0.2085.

The animals that were heavily infested by adult ticks were also heavily infested by nymphs (Figure 7). Indeed, there was a significant positive correlation between adult and nymph tick burdens (Pearson correlation = 0.390; *p* < 0.005). Moreover, there was a positive correlation between the individual infestation rankings for adult ticks and nymphs (Pearson correlation = 0.465; *p* < 0.001).

## Discussion

The control of *H. scupense* may be attempted using a number of different control options including manual tick removal, acaricide application and improvement of cattle enclosures to prevent the off-host tick seeking shelter. A good knowledge of the biology and the behaviour of the target tick within its environment remains an essential prerequisite for the development of any effective strategy of control based on the above measures as well as on potential new alternatives such as anti-tick vaccination [8, 16].

The present study aims to reliably describe the dynamics of infestation of cattle by ticks during the active season of *H. scupense*. Tick control measures were completely avoided during the study period; accordingly all the farmers were asked neither to treat nor to manually remove ticks infesting their cattle. The present study was implemented in traditional cattle farms, which were known to harbour high populations of the tick *H. scupense* [2, 9]. Furthermore, the timing of the study was chosen to fit with the seasonal activity of this tick species [3]. Other studies carried out in the same region have not shown any difference in tick dynamics except for the presence of an overlap between the adults and nymphs, which was reported in the present study for the first time in Tunisia [2, 9].

The majority of animals (94.2%) surveyed were infested by ticks (either adult or immature). Almost all the adult ticks collected from the sentinel cows (98.6%) were *H. scupense* as were all the adults emerging from nymphs forced to moult in the laboratory. Due to the previously established predominance of *H. scupense* in the targeted farms, these findings were expected [2, 9]. Consequently, it is reasonable to assume that the adult and immature ticks counted on cattle during the study period were also *H. scupense*.

The dynamics of tick activity recorded in the present study was in accordance with previous data on the seasonal activity of *H. scupense* [2, 20]. A single generation of ticks is annually observed; the adult ticks appeared between June and late November with a peak occurring during late June. Unfortunately no visits were made during May and this probably leads us to underestimate the tick population [3]. The female ticks detach from animals, lay eggs, which hatch to larvae that feed on cattle and moult on the cattle to become nymphs. The latter were observed from early September until late November and peaked in mid-October. As observed in other studies [3, 22], our results show that tick infestation is overdispersed, as 5% of the animals are infested by 16.72% and 52.53% of adults and nymphs, respectively. These hyper-attractive animals for ticks tend to remain the same during the period of activity of adult ticks and lesser of nymphs of *H. scupense*. The hyper-infested animals designated as “attractive for *H. scupense*”, as shown by Stachurski [22] in *Amblyomma* spp., play a major role in tick population dynamics and in the epidemiology of tick-borne diseases such as tropical theileriosis. Several hypotheses have been advanced for the presence of a discrepancy between individual tick burdens, such as the quantity of released attractants and body surface of individuals [21]. This discrepancy can also be due to a failure of attachment or ineffective feeding, due to the resistance of cattle to tick infestation. A combination of these putative causes in the same animal may also be possible. In the case of *H. scupense* this selective control approach could bring a better control of both adult and immature *H. scupense* ticks.

Furthermore, skewdness of ID in the animals was higher in calves than in adults, suggesting the existence of explanatory factors that influence the infestation intensity among calves. The curves of ID have an exponential or a triangular distribution for *H. scupense* ticks. Stachurski [22] observed a bell-shaped ID curve for *Amblyomma variegatum* distribution. Pegram et al. [17] demonstrated the presence of a correlation between tick counts and individuals when infested by *A. variegatum* and *Rhipicephalus appendiculatus*. In the absence of tick species variation, these authors suggested that selection of host resistance for all tick species is feasible. This specificity was maintained during all the survey period for both adults and nymphs. The presence of low ticks burdens in some animals (as observed in the two sentinel cows) is due to a resistance against ticks and/or a low attractiveness to them. It may be wise for the stockholder to identify and retain such animals leading to a decrease of tick biomass in the farm, and presumably a decrease of *T. annulata* infection risk.

As already established by previous works [2, 7], we found in this study a significant positive correlation between *H. scupense* adult tick burdens and the age of animals. Flach et al. [7] reported a positive correlation only between nymph infestation and animal age. The implication of this tick distribution is important in terms of *T. annulata* transmission, since older animals are expected to carry more parasite variants due to their exposure to repeated *T. annulata* infection [24].

Temperature and hygrometry are the most important abiotic factors influencing tick activity, their effects being expressed differently according to tick species [17]. Temperature determines tick development rate and may also effect survival and relative humidity is known to have an influence on tick survival [15, 21]. In our study, we noticed that adult tick activity is occurring during the summer season, corresponding to the hottest months with lowest hygrometry of the year (data not shown).

Predilection sites are known for some tick species, but to date these have not been established for *H. scupense*. For instance, adult *H. rufipes* Koch, 1844 attach preferentially to the anal region, whilst *H. truncatum* Koch, 1844 attach preferentially in the brush of hair at the tip of the tail, between the hooves, in the inguinal and perianal areas and in the scrotum and udder [11]. The present work shows that *H. scupense* adult ticks attach preferentially to the posterior udder quarters and in the lower part of the body, which concentrate 61.91% of adults and 68.92% of nymphs. Less exposed regions of the animals’ skin (receiving little air) are rich in carboxylic acid, phenol and indole end-products which are attractive to hard ticks [6]. The determination and quantification of the preferential attachment sites is of importance for the success of tick manual removal and acaricide use. This will improve the effectiveness of these control methods, decrease the quantity of acaricides used on animals and save time and labour. This is particularly important for nymphs as they are not easily seen. For instance, acaricide application to the udder and the inguinal region may eliminate 51.49% and 84.05% of adults and nymphs respectively. However, hand removal of ticks from the udder during milking may eliminate 48.76% and 68.74% of adults and nymphs respectively by this free and more environmentally friendly option.

The sex ratio was not found to be significant; the portions of females were lower than those of males in all the visits except one. This finding is unsurprising as males are more mobile than females and stay for a longer period on cattle. It was observed with the brown dog tick *R. sanguineus* (Latreille, 1806) that dogs infested by female ticks received more immigrant ticks than dogs initially infested by ticks of both sexes [13]. The dynamics of activity of males *versus* female ticks needs to be better studied since it may suggest novel control measures based on perturbation of tick mating.

In conclusion, the present study on the phenology of infestation of cattle by *H. scupense* provides basic but important data, which may be useful in optimising tick-borne disease control measures in the North African context. In particular, this information may be used to improve acaricide usage policy and will likely help underpin the development of novel approaches such as anti-tick vaccination.
